# Hydroxytyrosol Modulates Adipocyte Gene and miRNA Expression Under Inflammatory Condition

**DOI:** 10.3390/nu11102493

**Published:** 2019-10-17

**Authors:** Egeria Scoditti, Sara Carpi, Marika Massaro, Mariangela Pellegrino, Beatrice Polini, Maria Annunziata Carluccio, Martin Wabitsch, Tiziano Verri, Paola Nieri, Raffaele De Caterina

**Affiliations:** 1 National Research Council (CNR) Institute of Clinical Physiology (IFC), 73100 Lecce, Italy; egeria.scoditti@ifc.cnr.it (E.S.); marika.massaro@ifc.cnr.it (M.M.); maria.carluccio@ifc.cnr.it (M.A.C.); 2 Laboratory of Molecular Pharmacology, Department of Pharmacy, University of Pisa, 56126 Pisa, Italy; sara.carpi@unipi.it (S.C.); beatrice.polini@farm.unipi.it (B.P.); paola.nieri@farm.unipi.it (P.N.); 3 Laboratory of Applied Physiology, Department of Biological and Environmental Science and Technology (DISTEBA), University of Salento, 73100 Lecce, Italy; mariangela_pellegrino@yahoo.it (M.P.); tiziano.verri@unisalento.it (T.V.); 4 Division of Pediatric Endocrinology, Diabetes and Obesity, Department of Pediatrics and Adolescent Medicine, University of Ulm, 89075 Ulm, Germany; Martin.Wabitsch@uniklinik-ulm.de; 5 Cardiology Division, Pisa University Hospital, 56126 Pisa, Italy

**Keywords:** adipocyte, exosome, gene expression, hydroxytyrosol, inflammation, insulin resistance, extra virgin olive oil, miRNA, obesity, polyphenol

## Abstract

Chronic inflammation of the adipose tissue (AT) is a major contributor to obesity-associated cardiometabolic complications. The olive oil polyphenol hydroxytyrosol (HT) contributes to Mediterranean diet cardiometabolic benefits through mechanisms still partially unknown. We investigated HT (1 and 10 μmol/L) effects on gene expression (mRNA and microRNA) related to inflammation induced by 10 ng/mL tumor necrosis factor (TNF)-α in human Simpson–Golabi–Behmel Syndrome (SGBS) adipocytes. At real-time PCR, HT significantly inhibited TNF-α-induced mRNA levels, of monocyte chemoattractant protein-1, C-X-C Motif Ligand-10, interleukin (IL)-1β, IL-6, vascular endothelial growth factor, plasminogen activator inhibitor-1, cyclooxygenase-2, macrophage colony-stimulating factor, matrix metalloproteinase-2, Cu/Zn superoxide dismutase-1, and glutathione peroxidase, as well as surface expression of intercellular adhesion molecule-1, and reverted the TNF-α-mediated inhibition of endothelial nitric oxide synthase, peroxisome proliferator-activated receptor coactivator-1α, and glucose transporter-4. We found similar effects in adipocytes stimulated by macrophage-conditioned media. Accordingly, HT significantly counteracted miR-155-5p, miR-34a-5p, and let-7c-5p expression in both cells and exosomes, and prevented NF-κB activation and production of reactive oxygen species. HT can therefore modulate adipocyte gene expression profile through mechanisms involving a reduction of oxidative stress and NF-κB inhibition. By such mechanisms, HT may blunt macrophage recruitment and improve AT inflammation, preventing the deregulation of pathways involved in obesity-related diseases.

## 1. Introduction

Recent changes in human lifestyle, with a persistent positive energy balance, have resulted in a marked and alarming increase in the incidence of obesity, currently considered a worldwide health problem and a major risk factor—and hence a therapeutic target—for insulin resistance (IR), type 2 diabetes (T2DM) and cardiovascular disease (CVD) [1].

Abnormal adipose tissue (AT) expansion in obesity, coupled with hypoxia and adipocyte death, results in an aberrant adipocyte production of lipid mediators, reactive oxygen species (ROS), and bioactive protein mediators collectively called adipokines, such as peptide hormones, cytokines/chemokines, and prostaglandins, which may all be involved in the onset of obesity-associated local and systemic chronic, low-grade inflammation, and related IR and vascular dysfunction [2,3]. Increased expression of chemoattractant proteins, such as monocyte chemoattractant protein (MCP)-1 and C-X-C Motif Ligand-10 (CXCL-10), by hypertrophic adipocytes induces adipose tissue recruitment as well as infiltration and pro-inflammatory activation of immune cells (e.g., macrophages and lymphocytes) [4]. Secretory products, among which tumor necrosis factor (TNF)-α, of such cells further alter adipocyte biology by potently activating inflammatory signaling, including the transcription factor nuclear factor (NF)-κB pathway, and inducing an aberrant expression and release of factors involved in metabolic deregulation, angiogenesis, matrix remodeling and fibrosis, as well as pro-inflammatory, pro-atherogenic and pro-thrombotic adipokines [5]. These cell products, in concert, further exacerbate AT and systemic inflammation and IR [6,7,8].

Active molecular players in the regulation of obesity-related pathophysiological processes could be microRNAs (miRNAs), small noncoding functional RNAs that post-transcriptionally negatively regulate gene expression by directly binding to the target mRNAs and inhibiting mRNAs translation or stability [9]. miRNA expression patterns and function are tissue-specific. In the AT miRNAs actively participate in the regulation of adipogenesis, adipokine production, inflammation, and intercellular communication [10]. Furthermore, miRNAs can be packaged into adipocyte-secreted extracellular microvesicles called exosomes (30–100 nm in diameter), that mediate the regulatory crosstalk between adipocytes and other AT cell types, mostly macrophages [11], and between the AT and other key metabolic distal organs [12,13]. Upon inflammation, several miRNAs can be dysregulated and epigenetically mediate some obesity-associated derangements. An example of this are miR-34a, which induces the activation of pro-inflammatory macrophages in the AT [14] and is associated with obesity and diabetes [15], miR-155, which promotes adipocyte dysfunction and inflammation [16], and let-7c, which, on the opposite, exerts anti-adipogenic effects in 3T3-L1 cells [17] and suppresses inflammatory macrophage polarization [18].

Targeting adipocyte inflammation and immune cell crosstalk in AT is an attractive perspective for the treatment of obesity-related metabolic and CV diseases. By controlling gene expression and production of metabolites, nutrients hold a great promise in the prevention and treatment of obesity development and cardiometabolic outcomes.

The Mediterranean diet is recognized as one of the healthiest dietary patterns conferring benefits against CVD [19] and related conditions, such as diabetes, hypertension, and the metabolic syndrome [20]. Favorable effects of macro-and micro-components of the Mediterranean diet have been increasingly reported, with major contributions ascribed to Mediterranean diet phytochemicals including extra-virgin olive oil (EVOO) polyphenols [21], among which the most representative is the ortho-diphenolic compound hydroxytyrosol (2-[3,4-dihydroxyphenil]-ethanol, HT) [22]. Besides strong antioxidant properties exerted through both direct radical scavenging activity and genomic regulation of the antioxidant machinery [23], HT features potent anti-inflammatory, anti-thrombotic, and anti-atherogenic properties, improving endothelial dysfunction, hemostatic and lipid profiles, and decreasing inflammatory cell activation, thus exerting a major impact through gene expression regulation [24,25,26,27]. Beneficial effects of HT against metabolic, oxidative and inflammatory derangements have been reported in genetic and diet-induced animal models of hypercholesterolemia [28] and of the metabolic syndrome [29,30], as well as reductions (improvements) in IR in humans [31]. Although some studies in murine and human adipocytes have shown the ability of HT to modulate lipid content and adipogenic gene expression [32,33], and to promote mitochondrial biogenesis and function [34], data concerning HT effects in the context of human AT inflammation are scarce. We have recently documented that HT counteracts adipocyte dysfunction induced by TNF-α in human adipocytes, significantly preventing the suppression of adiponectin expression and release [35]. 

Against this background, we hypothesized that HT may modulate the obesity-associated adipocyte inflammatory response, oxidative stress, IR, and vascular dysfunction. We here therefore evaluated the effects of HT on the expression of genes and miRNAs involved in TNF-α and macrophage-induced inflammatory and dysmetabolic phenotype of human adipocytes, and explored underlying molecular mechanisms. We here demonstrate that HT can improve adipocyte dysfunction by beneficially regulating the expression of mRNAs and miRNAs in inflamed adipocytes.

## 2. Materials and Methods

### 2.1. Materials

HT (≥98% purity) was obtained from Cayman Chemicals (Ann Arbor, MI, USA). Recombinant human insulin was from Roche Diagnostics (Mannheim, Germany). All other chemicals were obtained from Sigma Aldrich (St. Louis, MO, USA), unless otherwise indicated. 

### 2.2. Cell Cultures and Treatments

For the current study, we used human Simpson–Golabi–Behmel syndrome (SGBS) preadipocytes, a physiologically relevant and well-established cell model system resembling human AT [36]. SGBS cells are characterized by a high capacity for adipogenic differentiation over many generations and functionally behave like human primary adipocytes. They have been widely used as a representative in vitro model for the study of obesity and the related processes of adipogenesis, metabolism, and inflammation, and to explore the effect of natural and pharmacologic compounds on adipocyte biology [35,36]. These cells were a generous gift of Prof. Martin Wabitsch (Division of Pediatric Endocrinology, Diabetes and Obesity, Department of Pediatrics and Adolescent Medicine, University of Ulm, Ulm, Germany, among the study co-authors), and were cultured and differentiated into mature adipocytes as previously described [35]. Briefly, SGBS cells were maintained in Dulbecco’s Modified Eagle Medium (DMEM)/F12 containing 10% fetal bovine serum (FBS) and 1% penicillin/streptomycin, 33 µmol/L biotin, and 17 µmol/L pantothenate. For experimental purposes, cells were plated and allowed to reach confluence before the addition of serum-free differentiation medium (DMEM/F12 with 25 nmol/L dexamethasone, 250 µmol/L 3-isobutyl-1-methylxanthine, 2 µmol/L rosiglitazone, 0.01 mg/mL human transferrin, 20 nmol/L insulin, 100 nmol/L cortisol, 0.2 nmol/L triiodothyronine, 33 µmol/L biotin, and 17 µmol/L pantothenate) for 4 days. Cell medium was then changed to an adipogenic medium (DMEM/F12 with 0.01 mg/mL human transferrin, 20 nmol/L insulin, 100 nmol/L cortisol, 0.2 nmol/L triiodothyronine, biotin, and pantothenate) for further 10 days. On day 15, >90% of these cells undergo complete differentiation into mature adipocytes, as assessed using Oil Red-O lipid staining and the expression of adipocyte-specific mRNAs, such as lipoprotein lipase, adipocyte fatty acid binding protein (FABP4), peroxisome proliferator-activated receptor (PPAR)-γ, and the glucose transporter (GLUT)-4. 

AT inflammation was mimicked by incubating fully differentiated SGBS cells with medium supplemented with the pro-inflammatory cytokine TNF-α at 10 ng/mL during 18 h. For HT treatment, stock solution (100 mmol/L) was prepared in 70% ethanol and then diluted in fresh medium at the time of the experiments. SGBS cells were incubated with 1 and 10 µmol/L HT for 1 h before stimulation with TNF-α. A pretreatment time of 1 h was chosen for HT in consideration of early pilot time-course studies, showing maximal anti-inflammatory efficacy of HT with a 1 h-pretreatment, suggesting the need for cell incorporation and/or biotransformation for HT to be effective in our experimental condition. Accordingly, 1 h pretreatment with HT before TNF-α inflammatory stimulation has been previously demonstrated to prevent adipocyte dysfunction as indexed by a beneficial regulation of adiponectin [35]. Untreated controls (CTL) were adipocytes only incubated in medium containing an appropriate volume of solvent, corresponding to the maximal concentration of HT used (<0.025% *v*/*v*). 

Human monocytoid THP-1 cells were obtained from the American Tissue Culture Collection (Rockville, MD, USA) and maintained in RPMI 1640 medium supplemented with 2 mmol/L glutamine, 100 mg/mL streptomycin, 100 IU/mL penicillin, and 10% FBS in a 5% CO_2_ humidified atmosphere at 37 °C. For the preparation of macrophage-conditioned medium (MacCM), THP-1 monocytes were differentiated into macrophages with 100 nmol/L phorbol-12 myristate 13-acetate (PMA) for 48 h. MacCM was collected after an additional 48 h of incubation in serum-free basal medium containing 0.5% bovine serum albumin (BSA) and cleared by centrifugation. MacCMs from 5 independently performed cultures were pooled and then used for experiments. For indirect co-culture experiments, SGBS adipocytes were pre-treated with 1 and 10 µmol/L HT for 1 h HT and then exposed to 1% MacCM or vehicle (serum-free THP-1 basal medium containing 0.5% BSA) in adipogenic medium for 5 h, after which fresh medium was added and incubated for a further 24 h. Cells and culture media were then collected and stored at −80 °C until the analysis.

### 2.3. RNA Isolation and Real-Time Quantitative Polymerase Chain Reaction

Total RNA was isolated by using the TRIzol reagent (Invitrogen, Carlsbad, CA, USA) according to the manufacturer’s protocol. For real-time quantitative polymerase chain reaction (qPCR), total RNA (2 µg) was converted into first-strand cDNA by using the High Capacity cDNA Reverse Transcription kit (Applied Biosystems, Monza, Italy). The qPCR was performed in a CFX Connect Real-Time PCR Detection System (Bio-Rad Laboratories, Milan, Italy) by using primer sequences or Taqman Gene Expression Assays for the indicated adipokines. All reactions were done in triplicate, and the amount of mRNA was calculated by the comparative critical threshold (C_T_) method. To account for possible variations related to cDNA input or the presence of PCR inhibitors, the endogenous reference gene ribosomal 18 S was simultaneously quantified for each sample, and data were normalized accordingly. Results are expressed as fold increase relative to unstimulated control (made = 1).

### 2.4. Measurement of Adipokines in Culture Media

Media were collected after TNF-α or MacCM treatment, centrifuged for 5 min, and stored at –20 °C until the analysis. Levels of adipokines in the culture medium were determined using the pertinent Quantikine Human enzyme-linked immunosorbent assay (ELISA) kit (R&D Systems, Minneapolis, MN, USA) according to the manufacturer’s instructions. Adipokine concentration was calculated from the standard curve, normalized to the total protein content, and expressed as percent of the unstimulated control.

### 2.5. Measurement of Prostaglandin(PG)E_2_ in Culture Media

PGE_2_ levels in the culture media were measured using a competitive enzyme immunoassay from Cayman Chemicals, according to the manufacturer’s instructions.

### 2.6. Cell Lysis and Western Blotting

After treatments, whole cell lysates were prepared using Cell Lytic M (Sigma) and separated using mini-protean TGX precast gels under reducing and denaturing condition (Bio-Rad Laboratories, Milan, Italy). Resolved proteins were transferred onto nitrocellulose sheets (Amersham, Freiburg, Germany), and the resulting membranes were saturated with 5% blocking agent (Amersham, Freiburg, Germany) in Tris-buffered saline (TBS, 20 mmol/L Tris, pH 7.6, 132 mmol/L NaCl)/0.1% Tween 20 for 1 h at room temperature. Blots were then incubated overnight at 4 °C with primary antibodies against endothelial nitric oxide synthase (eNOS) (Merck-Millipore, Darmstadt, Germany), cyclooxygenase (COX)-2 (Santa Cruz Biotechnology, Dallas, TX, USA), and β-actin (Sigma), followed by a horseradish peroxidase-conjugated secondary antibody (Santa Cruz Biotechnology, Dallas, TX, USA). An enhanced chemiluminescence (ECL) kit (Amersham, Freiburg, Germany) was used to reveal positive bands, according to manufacturer’s instructions. Bands were analyzed quantitatively using the Image Studio Lite 4.0 software (LI-COR Inc., Lincoln, NE, USA) and normalized to β-actin levels.

### 2.7. Preparation of Nuclear Extracts and Measurement of NF-κB p65 DNA Binding Activity

SGBS adipocytes were pretreated with HT for 1 h and stimulated with 10 ng/mL TNF-α for 1 h. Nuclear proteins were extracted using the Nuclear Extract kit (Active Motif, Carlsbad, CA, USA) according to the manufacturer’s protocol. The activation of NF-κB was assessed using the ELISA-based TransAM NF-κB p65 kit (Active Motif, Carlsbad, CA, USA), following the manufacturer’s protocol. Briefly, the NF-κB TransAM kit contains a 96-well plate with immobilized oligonucleotides encoding an NF-κB consensus site (5′-GGGACTTTCC-3′). The active form of NF-κB contained in the nuclear cell extracts specifically binds to the oligonucleotide. The primary antibody used to detect NF-κB recognizes an epitope on p65 that is accessible only when NF-κB is activated and bound to its target DNA sequence. A horseradish peroxidase-conjugated secondary antibody provides a sensitive colorimetric readout that is quantified by spectrophotometry at 450 nm, with a reference wavelength set to 655 nm. Data are expressed as percent of unstimulated control.

### 2.8. Measurement of Intracellular ROS Production

SGBS adipocytes were pretreated with HT for 1 h and stimulated with 10 ng/mL TNF-α for 1 h in phenol red-free DMEM. After washing, cells were incubated with 5 μmol/L carboxy-H2DCFDA (Molecular Probes, a brand of ThermoFisher Scientific, Waltham, MA, USA) for 30 min at 37 °C. Fluorescence intensity were measured at an excitation wavelength of 488 nm and an emission wavelength of 530 nm on a fluorimeter (Fluoroskan II, Labsystem, Helsinki, Finland), and normalized to total protein content.

### 2.9. Exosome Isolation from Cell Culture Supernatants

Exosomes were isolated from cell culture conditioned supernatants using the protocol published by Lobb et al. [37]. Conditioned culture media were harvested from adipocytes and centrifuged at 300× *g* at 4 °C for 10 min to remove detached cells. Then, supernatants were filtered through 0.22 µm filters (Merck Millipore, Darmstadt, Germany) to remove contaminating apoptotic bodies, microvesicles and cell debris. Clarified conditioned culture media were then centrifuged in a Sorvall^TM^ MTX 150 micro-ultracentrifuge (Thermo Scientific) at 100,000× *g* at 4 °C for 90 min to pellet exosomes. The supernatant was carefully removed, and pellets containing exosomes were resuspended in 1 mL of ice-cold PBS. A second round of ultracentrifugation under the same condition was carried out, and the resulting exosome pellet resuspended in 200 µL of PBS.

### 2.10. Evaluation of miRNA Expression

The miRNeasy Mini kit (Qiagen, Hilden, Germany) was used for the purification and extraction of miRNAs from exosomes isolated from cell culture conditioned supernatants or adipocytes. The retro-transcription of extracted miRNAs was performed by using the miScript Reverse Transcription kit (Qiagen). The cDNA obtained was diluted 1:3 in RNase-free water from adipocytes, while the exosome-cDNA was used without dilution. The qPCR experiments were performed with the miScript SYBR-Green PCR kit (Qiagen), as previously reported [38]. Signals were detected on the MiniOpticon CFX 48 real-time PCR Detection System (Bio-Rad, Hercules, CA, USA). MiScript Primer Assays specific for hsa-miR-34a-5p (MIMAT0000255), hsa-miR-155-5p (MIMAT0000646) and hsa-let-7c-5p (MIMAT0000064), hsa-SNORD6 and hsa-RNU6 were obtained from Qiagen. MiRNA expression was calculated using the C_T_ method and normalized to the expression of housekeeping genes SNORD6 for adipocyte-derived miRNAs, and *Caenorhabditis elegans* miR-39 (Cel-miR-39) for exosome-derived miRNAs (exo-miRNAs).

### 2.11. Statistical Analysis

Results are expressed as means ± SD of at least 3 independent experiments performed in triplicate. We used the Student’s *t* test to compare means between control group and compound-treated group. We performed multiple comparisons by one-way analysis of variance (ANOVA). We considered a *p* level < 0.05 as statistically significant.

## 3. Results

### 3.1. HT Modulates TNF-α-Stimulated Gene Expression in Adipocytes

To investigate the protective effects of HT on TNF-α–induced inflammation in human adipocytes, SGBS cells were exposed to 1 and 10 µmol/L HT for 1 h and then stimulated with 10 ng/mL TNF-α for 18 h to induce inflammatory gene expression and protein secretion. 

**Figure 1:**
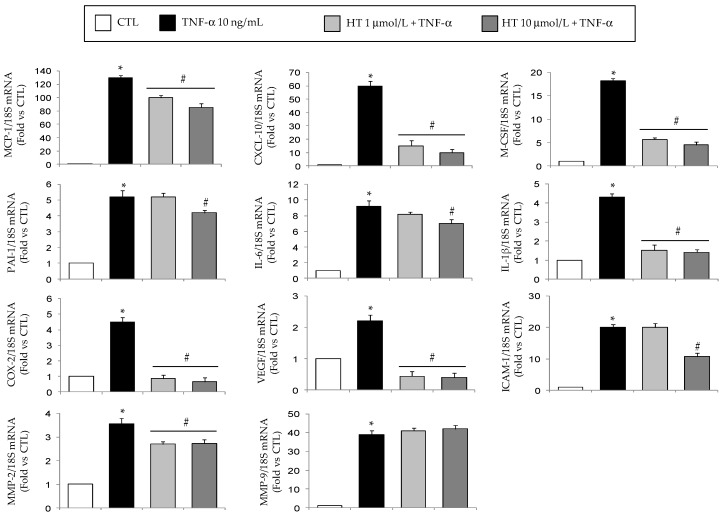
Modulation by HT of mRNA expression levels of genes associated with adipocyte inflammation. Simpson–Golabi–Behmel syndrome (SGBS) adipocytes were pretreated with HT (1 h) at the concentrations indicated, and then treated with 10 ng/mL TNF-α for 18 h. Total RNA was extracted from cells, and mRNA levels of the indicated genes were measured by qPCR using specific primers and probes and normalized to 18S RNA. Data (means ± SD, *n* = 3) are expressed as fold induction over untreated control (CTL). *p < 0.05 vs. CTL. #*p <* 0.05 vs. TNF-α alone.

Already at 1 µmol/L HT significantly (*p* < 0.05) prevented the TNF-α-induced upregulation of mRNA levels of MCP-1, CXCL-10, macrophage colony-stimulating factor (M-CSF), interleukin (IL)-1β, vascular endothelial growth factor (VEGF), COX-2, and metalloproteinase (MMP)-2, except for MMP-9 (Figure 1). HT also inhibited, at 10 µmol/L, the TNF-α-stimulated mRNA induction of IL-6, plasminogen activator inhibitor (PAI)-1, intercellular adhesion molecule (ICAM)-1, without any effect on MMP-9 mRNA levels (Figure 1). 

**Figure 2:**
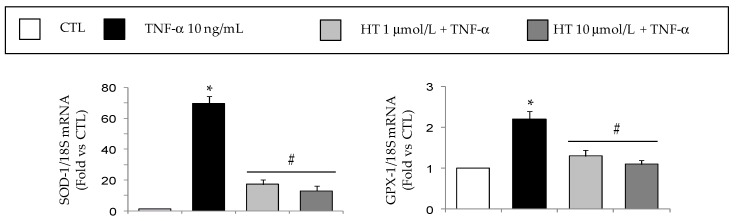
Modulation by HT of mRNA expression levels of genes associated with antioxidant response. SGBS adipocytes were pretreated with HT (1 h) at the concentrations indicated, and then treated with 10 ng/mL TNF-α for 18 h. Total RNA was extracted from cells, and mRNA levels of SOD-1 and GPX-1 were measured by qPCR using specific primers and probes and normalized to 18S RNA. Data (means ± SD, *n* = 3) are expressed as fold induction over untreated control (CTL). *p < 0.05 vs. CTL. #*p <* 0.05 vs. TNF-α alone.

Furthermore, HT attenuated the increase of superoxide dismutase (SOD)-1 and glutathione peroxidase (GPX) mRNA levels in response to TNF-α (Figure 2). 

**Figure 3:**
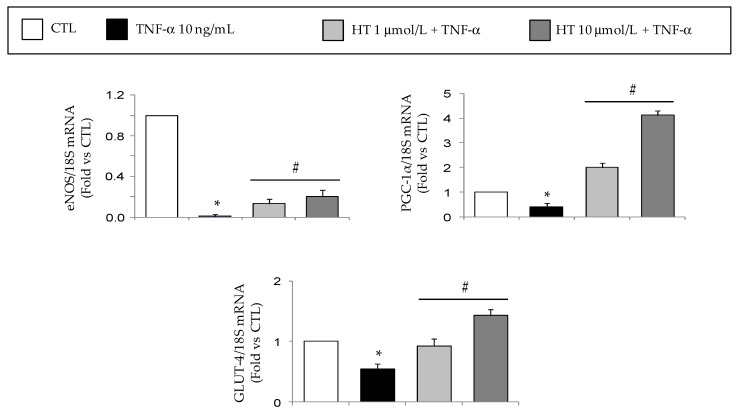
Modulation by HT of mRNA expression levels of genes associated with adipocyte metabolism and mitochondrial biogenesis. SGBS adipocytes were pretreated with HT (1 h) at the concentrations indicated, and then treated with 10 ng/mL TNF-α for 18 h. Total RNA was extracted from cells, and mRNA levels of eNOS, PGC-1α, and GLUT-4 were measured by qPCR using specific primers and probes and normalized to 18S RNA. Data (means ± SD, *n* = 3) are expressed as fold induction over untreated control (CTL). *p < 0.05 vs. CTL. #*p <* 0.05 vs. TNF-α alone.

Under inflammatory condition as induced by TNF-α, reduced mRNA gene expression of eNOS, peroxisome proliferator-activated receptor coactivator 1 alpha (PGC-1α), and GLUT-4 was observed (Figure 3), but HT concentration-dependently prevented this suppression restoring the expression levels of eNOS, PGC-1α, and GLUT-4. 

As previously reported [35], no measurable signs of HT cytotoxicity were noted at the time and the concentrations used (e.g., no floating cells, no visual differences in the number of attached cells or protein concentrations, and no abnormal changes in cell morphology) (not shown).

### 3.2. HT Modulates Macrophage Conditioned Medium-Stimulated Gene Expression in Adipocytes

Next, in order to more pathophysiologically mimic the low-grade inflammation in the obese AT, we reproduced the paracrine interaction between macrophages and adipocytes, that is implicated in inducing adipocyte dysfunction. To this aim, SGBS adipocytes were exposed to MacCM as a pro-inflammatory stimulus instead of TNF-α alone, and changes in the mRNA expression of several target genes were analyzed by qPCR. In this model system, we examined whether HT was able to counteract inflammatory changes in adipocytes induced by macrophage-secreted factors including, but not limited to, TNF-α. 

**Figure 4:**
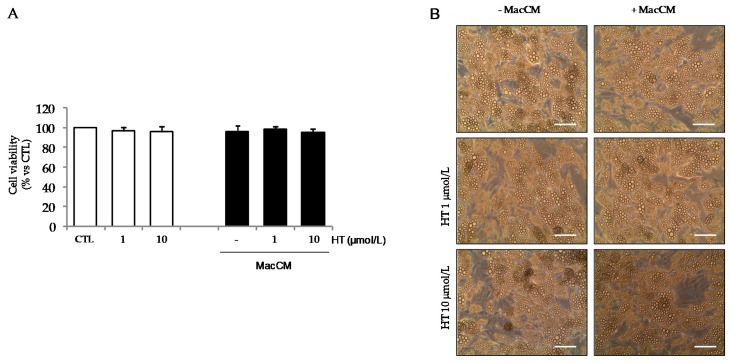
The effect of macrophage-derived conditioned medium treatment on adipocyte viability. SGBS adipocytes were pretreated with HT (1 h) at the concentrations indicated, and then either treated with macrophage-derived conditioned medium (MacCM) (black-filled bars), or left untreated (open white bars), for 18 h. (**A**) Cell viability was assessed by the MTT assay, and expressed as percent of untreated control (CTL). Data are means ± SD (*n* = 3). In (**B**), representative phase-contrast images of SGBS cells after treatments are shown. Scale bar = 50 μm.

Preliminary pilot studies in which SGBS adipocytes were incubated with medium supplemented with increasing concentrations of MacCM (1% to 10%) revealed that already 1% MacCM significantly increased the expression of inflammatory genes by adipocytes, as previously reported [39]. This effect occurred in the absence of changes in the adipocyte viability (Figure 4A) and lipid content, as judged morphologically (Figure 4B).

**Figure 5:**
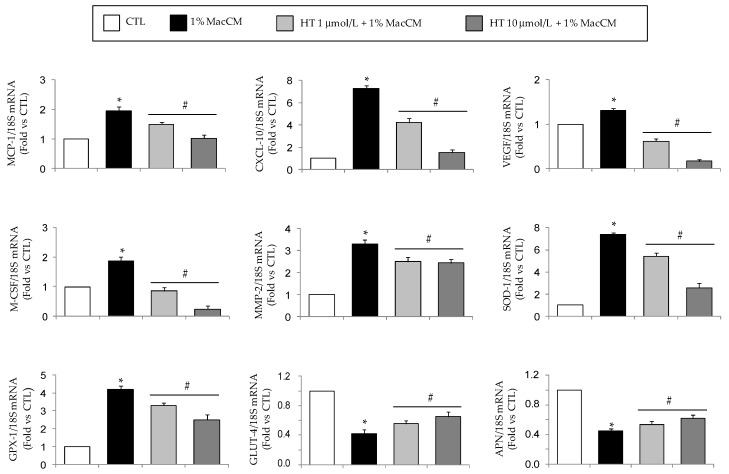
Effect of HT on macrophage conditioned medium-induced changes in adipocyte gene expression. SGBS adipocytes were pretreated with HT (1 h) at the concentrations indicated, followed by incubation with 1% RPMI 1640 medium (CTL) or macrophage-derived conditioned medium (MacCM) as described in Methods. Total RNA was extracted from cells, and mRNA levels of the indicated genes were measured by qPCR using specific primers and probes, and normalized to 18S RNA. Data (means ± SD, *n* = 3) are expressed as fold induction over untreated control (CTL). *p < 0.05 vs. CTL. #*p <* 0.05 vs. TNF-α alone.

Therefore, we conducted further experiments with 1% MacCM. As shown in Figure 5, MacCM caused a robustly enhanced MCP-1, CXCL-10, VEGF, M-CSF, MMP-2, SOD-1, and GPX mRNA expression in adipocytes, while GLUT-4 and adiponectin expressions were reduced. HT pre-treatment resulted in a concentration-dependent reduction of MacCM-stimulated MCP-1, CXCL-10, VEGF, M-CSF, MMP-2, SOD-1, and GPX mRNA expression, and an attenuation of MacCM-stimulated decrease of GLUT-4 and adiponectin when compared to MacCM treatment alone. Therefore, the inflammatory microenvironment mimicked by MacCM induced a shift toward a pro-inflammatory and dysmetabolic gene expression profile in adipocytes, that was significantly reverted by HT.

### 3.3. HT Modulates TNF-α-Induced Changes in Secreted or Intracellular Adipokines in Adipocytes

To confirm that HT modulation of the target genes at the mRNA levels resulted in concordant changes in the corresponding secreted or intracellular protein levels, the mRNA expression data for a subset of genes differentially regulated by HT were confirmed measuring protein release in the cell culture medium by immunoassays or intracellular protein levels by Western analysis, as appropriately.

**Figure 6:**
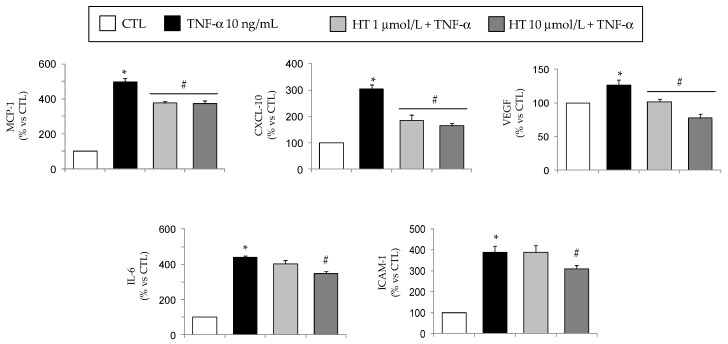
Attenuation by HT of TNF-α-induced protein release of adipokines in human adipocytes. SGBS adipocytes were pretreated with HT (1 h) at the concentrations indicated, and then treated with 10 ng/mL TNF-α for 18 h. Adipokine levels in the culture medium and ICAM-1 surface expression were determined by ELISA, and expressed as percent of untreated control (CTL). Bars represent means ± SD (*n* = 3). *p < 0.05 vs. CTL. #*p <* 0.05 vs. TNF-α alone.

At ELISA, we found increased levels of MCP-1, CXCL-10, VEGF, and IL-6 in the culture media in response to TNF-α, and an attenuation by HT in agreement with qPCR data (Figure 6). Furthermore, HT downregulated TNF-α-induced cell surface protein expression of ICAM-1 (Figure 6).

**Figure 7:**
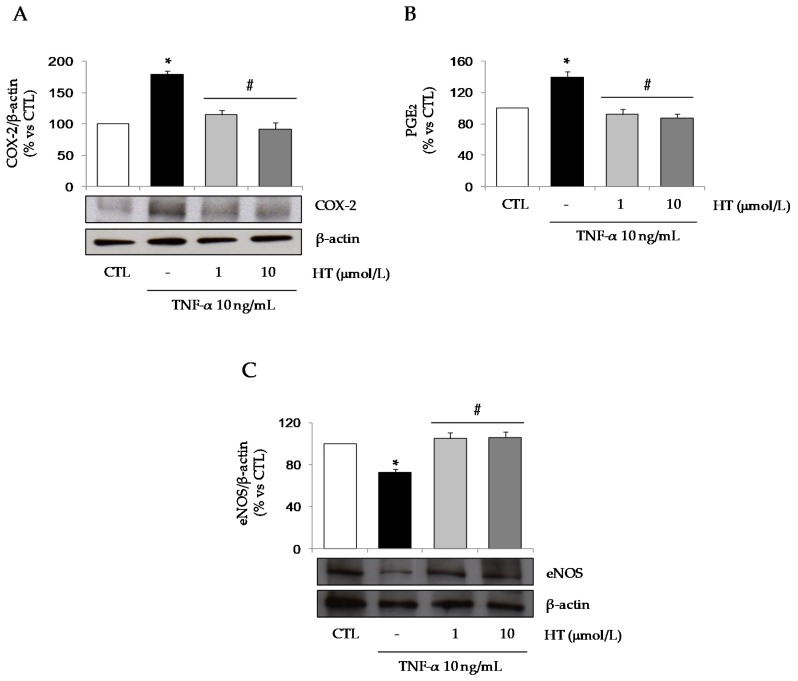
Effect of HT on the intracellular levels of COX-2 and eNOS. SGBS cells were treated with 1 and 10 μmol/L HT and then stimulated with 10 ng/mL TNF-α for 18 h. COX-2 (**A**) and eNOS (**C**) intracellular protein levels were determined by Western analysis using specific antibodies. COX-2 and eNOS band intensities were normalized to β-actin, and are expressed as percent of untreated control (CTL). (**B**) Cell culture media were collected to assess the production of COX-2 product PGE_2_. Data are means ± SD (n = 3). *p < 0.05 vs. CTL. #*p <* 0.05 vs. TNF-α alone.

At Western analysis, TNF-α caused a significant induction of COX-2 protein expression and a corresponding release of the COX-2 inflammatory product PGE_2_ in the culture medium, that were completely prevented by HT (Figure 7A,B). Concordantly, HT also restored the reduced protein expression of eNOS as induced by TNF-α (Figure 7C). 

### 3.4. HT Prevents TNF-α-Induced ROS Production and NF-κB Activation

In search of the upstream site of interference by HT with the signaling pathway(s) mediating modulation of inflammatory gene expression in adipocytes, and given the role of the redox-sensitive transcription factor NF-κB in orchestrating inflammatory transcriptional programs in adipocyte dysfunction [40], we examined the HT effect on intracellular ROS production and the consequent activation of NF-κB under TNF-α stimulation.

**Figure 8:**
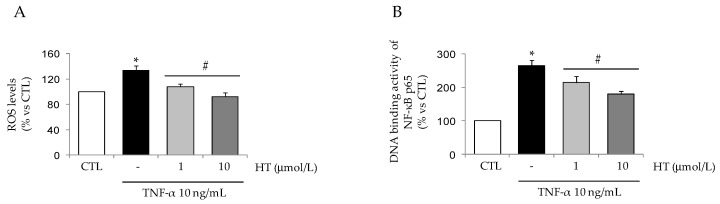
Attenuation by HT of TNF-α-induced ROS production and NF-κB activation. SGBS cells were treated with 1 and 10 μmol/L HT and then stimulated with 10 ng/mL TNF-α for 1 h. (**A**) Intracellular levels of ROS were monitored by using the redox sensitive fluorescent probe carboxy-H2DCFDA, and expressed as percent of unstimulated control (CTL). (**B**) Nuclear proteins were analyzed for NF-κB p65 DNA-binding activity by ELISA as described in Methods. Data are expressed as percent of untreated control (CTL). Bars represent means ± SD (*n* = 3). *p < 0.05 vs. CTL. #*p <* 0.05 vs. TNF-α.

Basal ROS production, as assessed by dichlorofluorescein fluorescence, was significantly increased— by about 35%—by TNF-α (Figure 8A). Pre-incubation of adipocytes with 1 and 10 µmol/L HT before cytokine stimulation almost completely abrogated the induced production of ROS. Correspondingly, HT was able to prevent TNF-α-induced activation of NF-κB as measured by the DNA binding activity of the NF-κB p65 subunit (by 20% ± 5% for 1 µmol/L HT and 30% ± 7% for 10 µmol/L HT compared with TNF-α alone) (Figure 8B).

### 3.5. HT Modulates TNF-α-Induced Inflammation-Linked miRNA Expression in Adipocytes and in Adipocyte-Derived Exosomes

**Figure 9:**
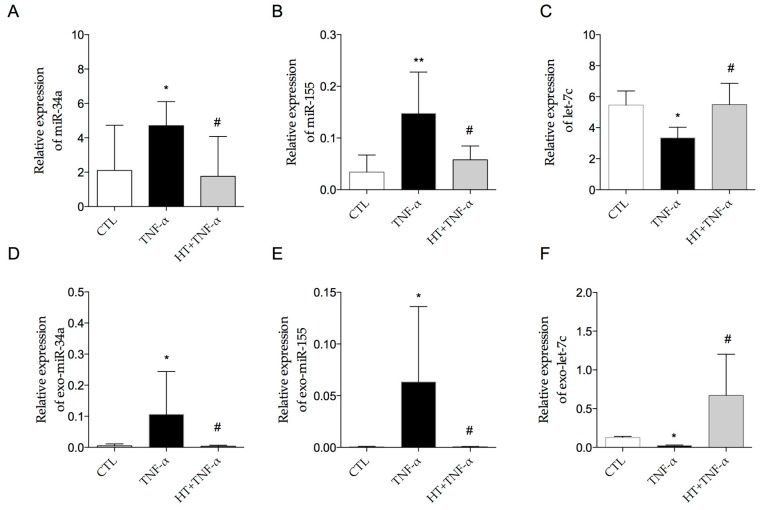
Modulation by HT of TNF-α-induced miRNA expression changes in adipocytes and in exosomes isolated from conditioned media. SGBS cells were treated with 10 μmol/L HT, and then stimulated with 10 ng/mL TNF-α for 18 hours. Total miRNAs were extracted from cells, and miR-34a (**A**), miR-155 (**B**) and let-7c levels (**C**) were measured by qPCR, analyzed using the Ct method, and normalized to SNORD6 levels. Total miRNAs were extracted from exosomes (exo-miR), and miR-34a (**D**), miR-155 (**E**), and let-7c (**F**) levels were measured by qPCR, analyzed using the Ct method, and normalized to Cel-miR-39. Bars represent means ± SD (*n* = 3). *p < 0.05 versus CTL. **p < 0.01 vs. CTL. # *p* < 0.05 vs. TNF-α.

Under inflammatory conditions as induced by TNF-α, we observed an increased expression of miR-34a (4.706 ± 1.399 vs. 2.101 ± 2.629 in adipocytes and 0.106 ± 0.139 vs. 0.004 ± 0.003 in exosomes) (Figure 9A,D) and miR-155 (0.148 ± 0.078 vs. 0.034 ± 0.033 in adipocytes and 0.063 ± 0.072 vs. 0.0005 ± 0.0005 in exosomes) (Figure 9B,E). On the contrary, the inflammatory stimulus decreased let-7c expression levels (3.347 ± 0.683 vs. 5.459 ± 0.910 in adipocytes and 0.022 ± 0.007 vs. 0.125 ± 0.016 in exosomes) (Figure 9C,F). 

Pre-treatment with HT significantly (*p* < 0.05) prevented the TNF-α-induced upregulation of miR-34a and miR-155 levels as well as the down-regulation of let-7c levels in both cells (Figure 9A–C) and exosomes (Figure 9D–F).

## 4. Discussion

We here investigated the gene expression profile of prototypical markers of obesity-associated dysfunction in human inflamed adipocytes under treatment with HT, the most representative simple phenol of EVOO. We found that HT, in a nutritionally relevant concentration range (1 to 10 µmol/L) attainable after EVOO consumption, significantly prevents the TNF-α- and macrophage secreted factors-induced inflammatory and dysmetabolic phenotype of adipocytes. This occurs by beneficially counter-regulating the disturbed pattern of mRNA expression of chemokines/cytokines (MCP-1, CXCL-10, M-CSF, IL-1β, IL-6), angiogenic growth factors (VEGF), inflammatory and proteolytic/remodeling enzymes (COX-2, MMP-2), adhesion molecules (ICAM-1), thrombogenic factors (PAI-1), antioxidant enzymes (SOD, GPX), metabolic effectors (eNOS, PGC-1α, GLUT-4), as well as the expression of inflammation-related miRNAs (miR-34a, miR-155, let-7c). These effects were associated with—and plausibly mediated by—a reduced production of ROS and the modulation of NF-κB activation. Although we confirmed at the protein level only some selected transcripts of the inflammatory markers (MCP-1, CXCL-10, VEGF, IL-6, ICAM-1), we always demonstrated concordant changes of mRNA and protein expression under HT.

AT is a major target for the prevention and treatment of cardiometabolic diseases, through the modulation of adipocyte differentiation, adipokine secretion, inflammation and insulin sensitivity. Adipocyte dysfunction is featured by derangements in AT cellularity, with excessive lipid storage, adipocyte hypertrophy and immune cell infiltration, as well as altered inflammatory and metabolic functions [8]. AT inflammation is exacerbated by the increased infiltration and activation of macrophages and other immune cells, whose direct contact with adipocytes and with secretory products, including TNF-α, potently stimulates inflammatory responses and a metabolic dysregulation in adipocytes, with a shift in adipocyte secretory patterns from a less inflammatory profile to a predominately pro-inflammatory profile. This is in part responsible for the development of AT and systemic IR and related CV outcomes [7,41]. Therefore, restoring the balance between pro- and anti-inflammatory adipokines is a potential therapeutic strategy to treat or prevent obesity-related comorbidities. A crucial aspect of AT inflammation in obesity is the phenotypic switch of resident macrophages from the alternatively activated, anti-inflammatory M2 polarization state, important for the proper tissue function and homeostasis, to the classically activated, pro-inflammatory M1 polarization state, able of producing factors that instigate chronic metabolic inflammation and IR [42]. Therefore, the inflammatory paracrine loop between adipocytes and macrophages should be regarded as an attractive target to attenuate inflammation-associated outcomes in obesity. 

To model macrophage and adipocyte paracrine interactions in vitro, we exposed adipocytes to MacCM, containing a mixture of pro-inflammatory factors. Correspondingly, MacCM-treated adipocytes increased the transcription of inflammation-related genes, including chemokines (MCP-1, M-CSF, CXCL-10), MMP-2, VEGF, antioxidant enzymes (SOD, GPX), and downregulated the mRNA levels of GLUT-4 and adiponectin. Similar to the effects exerted in TNF-α-stimulated cells, HT treatment attenuated the MacCM-induced increased expression of detrimental effectors, while partially recovering GLUT-4 and adiponectin expression, thus suggesting an effective anti-inflammatory action of HT in obesity-mimicking inflammatory conditions.

Although a great deal of research has been devoted to characterizing HT role in oxidative stress resistance, inflammation, cancer and atherosclerosis [22], only recent studies have explored its effects in obesity and T2DM [43]. Human studies suggest anti-diabetic and anti-obesity effects of EVOO dietary supplementation [44,45,46], an HT nutraceutical [47], and an olive leaf extract rich in HT [31,48]. Chronic supplementation with olive leaf polyphenols, including HT, improved parameters of glucose homeostasis, such as plasma insulin and glycated hemoglobin in T2DM patients [48], and pancreatic β-cell secretory capacity and insulin sensitivity in overweight men [31]. Concordantly, some animal studies have reported anti-obesity effects [30,49], as well as improvement in systemic metabolic parameters (IR, hyperglycemia, hyperlipidemia) [28,30,50] and AT distribution [28,29] after supplementation with HT, olive leaf extract rich in HT, or EVOO. These effects are consistent with the in vivo observed beneficial modulation of the expression of genes related to insulin sensitivity and the metabolic syndrome in human peripheral blood monocytes by EVOO [51], and related to adipogenesis (downregulation of PPARγ, CCAAT/enhancer-binding protein (C/EBP)α, CD36, fatty acid synthase, and leptin), and mitochondrial biogenesis (upregulation of mitochondrial transcription factor A (ATFAM), nuclear respiratory factor (NRF)-1, and cytochrome C oxidase subunit 2) coupled with thermogenesis (upregulation of SIRT1, PGC-1α, and uncoupling protein (UCP)-1) in mice AT by olive leaf extract [49]. 

Regarding the direct role of HT in adipocytes, in vitro reports have documented the inhibition by HT of murine and human adipogenic differentiation accompanied by stimulation of mitochondrial biogenesis and function, increased lipolysis [32,33] and fatty acid oxidation and energy expenditure [34]. These effects are coupled with beneficial regulation of expression of genes associated with adipogenesis, mitochondrial function and metabolic control, including SREBP-1c, GATAs, SIRT1, PGC-1α, carnitine palmitoyltransferase (CPT) 1, cannabinoid CB1receptor, PPARα and PPARγ [33,34,52]. In addition to improvement of adipocyte function via its anti-adipogenic activity, HT has been reported to beneficially regulate the expression of the insulin-sensitizing and anti-inflammatory adipokine adiponectin, as well as the leptin-to-adiponectin ratio in human adipocytes under TNF-α-induced inflammation [35]. Moreover, beneficial dowregulation of the pro-inflammatory adipokine visfatin has been observed after 8-week consumption of EVOO compared to refined olive oil in patients with T2DM [45].

In the light of HT effects on AT energy metabolism, and in order to gain more insight into the potential modulation by HT of the obesity-associated inflammatory response and metabolic dysregulation, in the present study we analyzed an obesity-related gene expression profile in HT-treated human hypertrophic adipocytes under inflammatory condition. As far as we know, this is the first time that this issue has been addressed.

A key original finding from the present study is the demonstration that HT significantly inhibits TNF-α-induced expression of the chemokines/cytokines MCP-1, CXCL-10, and M-CSF by human adipocytes. Increased production of chemokines in obese AT has been implicated in the regulation of monocyte recruitment to AT [53]. Macrophage infiltration and inflammation-related gene expression in the AT precedes the development of IR in animal models [53,54]. AT macrophage infiltration also correlates with systemic IR in obese human subjects [55]. Supportively, inhibition of macrophage infiltration into obese AT, through genetic and/or pharmacologic strategies, improved the dysregulation of adipocytokine production and ameliorated obesity-induced inflammation and IR, thus providing a therapeutic target for the metabolic syndrome [4,56].

Chemokines can be classified into two major subfamilies according to amino acid sequence: the C-C motif ligand (CCL) and the C-X-C motif ligand (CXCL) subfamilies. Among the chemokines produced by adipocytes, MCP-1, also known as CCL2, is a potent chemoattractant specific for monocytes and macrophages. MCP-1 is produced by both fractions of AT, i.e., mature adipocytes and stromal vascular cells including resident macrophages. The involvement of the MCP-1/chemokine receptor 2 (CCR2) pathway has been extensively studied as the mechanism underlying macrophage infiltration into obese AT [4,56]. Circulating and AT levels of MCP-1 and levels of CCR2-expressing inflammatory cells are increased in obesity, and are strongly associated with IR [57,58]. MCP-1 is also crucially involved in monocyte/macrophage recruitment into the arterial wall, a major process leading to atherosclerosis [59], thus providing a possible causative link between obesity and related complications, such as CVD. Several reports have suggested the role of other chemotactic factors in obesity-induced macrophage infiltration and IR, including CXCL-10 or interferon-γ-inducible protein 10 (IP-10), which is a potent chemoattractant for various leukocyte subsets. CXCL-10 is produced by mature human adipocytes from various depots, with a positive correlation with body mass index (BMI), visceral adiposity, other parameters of obesity and the risk of T2DM [60], and has also been implicated in the pathogenesis of atherosclerosis as well as obesity-related inflammation [61]. M-CSF (or CSF1) is the primary regulator of macrophage development and survival. Interestingly, Weisberg et al. [53] demonstrated the dependence of AT macrophage recruitment on M-CSF, given that macrophage-deficient Csf1 knockout mice are also deficient in F4/80 (a macrophage-specific antigen) expressing cells in AT. 

With increasing adiposity, AT may release signals such as MCP-1 and CXCL-10, causing increased monocyte influx. The production of M-CSF by adipocytes may then create a permissive microenvironment for these monocytes to differentiate and survive as mature AT macrophages. The blockade/reduction of chemotactic factor gene expression in adipocytes by HT may disrupt immune cell chemotaxis and activation and reduce the downstream effects of obesity-induced inflammation. Previous studies have shown the ability of HT or its metabolites to reduce the expression/release of MCP-1 by human vascular endothelial cells [62], human monocytes [27], murine macrophages [24], and visceral AT of hypercholesterolemic rats [28], as well as to decrease LPS-stimulated CXCL-10 mRNA and protein expression by murine macrophages [24]. Noteworthy, ours is the first report of HT modulation of MCP-1, CXCL-10 and M-CSF gene expression in human adipocytes, and points to novel molecular targets of HT for potentially blocking macrophage recruitment in the obese AT.

We also showed here that HT inhibits TNF-α-stimulated expression of pro-inflammatory cytokines including IL-6 and IL-1β. In agreement with our findings, HT also impaired cytokines gene and protein expression in activated murine macrophages, although at higher concentrations (>10 µmol/L) [24]. Obesity is strongly associated with high AT and circulating levels of both cytokines, with AT—mainly in the visceral compartment—as a major contributor to their systemic levels [63]. Both cytokines play a major role in inducing obesity-associated IR, as well as local and systemic inflammation [64,65]. The involvement of pro-inflammatory cytokines including IL-6 and IL-1β in an autocrine/paracrine stimulatory loop, via the upregulation of their own expression and the expression of chemokines including MCP-1 and other inflammatory factors by adipocytes, supports a vicious cycle of perpetuating AT inflammation and immune cell infiltration. Direct pro-atherogenic effects of these cytokines/chemokines on the vascular system [3] coupled with the IL-6-induced hepatic production of acute-phase reactants such as C-reactive protein (CRP), a known CV risk marker, provide potential mechanistic links between obese AT and its systemic sequelae, such as chronic inflammation, IR and CVD. Therefore, chemokines and cytokines downregulation by HT in inflamed adipocytes is a novel finding that may have a major impact on obesity-related inflammation and dysmetabolism.

PAI-1 is an adipokine with pro-inflammatory and pro-thrombotic action, which plays a role in cell migration, angiogenesis, and tissue remodeling. Plasma PAI-1 levels are remarkably enhanced in humans with obesity/IR, T2DM, and CVD, with the AT as a major source of elevated PAI-1 levels [66]. PAI-1 deficiency reduces adiposity, and attenuates diet-induced obesity and IR in animal models [67]. The ability of HT (at 10 µmol/L) to reduce PAI-1 expression by inflamed adipocytes is consistent with what reported for other polyphenols [39]. The beneficial role of EVOO consumption in reducing plasma levels of PAI-1 has been previously shown [68], and our results suggest a potential contribution by HT to this effect, with implications for preventing obesity-associated atherothrombotic vascular disease.

Other upregulated genes in obesity encode surface adhesion molecules that may help promoting leukocyte infiltration and retention in the tissue. Among adhesion molecules, ICAM-1 is a member of the immunoglobulin superfamily, and is mainly expressed on a wide variety of cells under conditions of inflammation to function as a leukocyte adhesion receptor [69]. Although one of the best documented roles of ICAM-1 is that of a mediator of leukocyte recruitment during atherosclerotic plaque formation, also being a marker, in its soluble form, of endothelial dysfunction and inflammation, its role has expanded to the inflammatory response in obesity and the AT. Accordingly, increased circulating levels of soluble ICAM-1 have been found in obese subjects, and are positively correlated with central adiposity and IR [70]. Furthermore, elevated expression of ICAM-1 has been described in the abdominal AT of mice with diet-induced obesity [71], and in the visceral AT of obese women, in correlation with the expression of the macrophage-related marker CD68 and with BMI [72]. Therefore, obese AT expresses adhesion molecules including ICAM-1, and may be a potential source of circulating adhesion molecules in obesity. Although vascular endothelial cells and other cells within the obese AT may contribute to the increased adhesion molecules expression, surface expression of ICAM-1 in adipocytes may also be involved, because adipocyte ICAM-1 is inducible by cytokines as well as by macrophage-conditioned media [6], and mediates leukocyte-adipocyte adherence [73]. Besides reducing cytokine/chemokine gene expression in inflamed adipocytes, and in line with its ability to dampen inflammatory adhesion molecule expression in other cells types such as endothelial cells [74], HT could attenuate the TNF-α-stimulated mRNA and protein expression of ICAM-1 in adipocytes, thus targeting another key event in the initiation and maintenance of the inflammatory cascade in obesity.

We also observed that HT decreased the inflammatory induction of active players—and potential targets—of obesity-related remodeling, including the proteolytic enzyme MMP-2 (gelatinase A), the proangiogenic VEGF-A, as well as the pro-inflammatory enzyme COX-2 and its eicosanoid product PGE_2_. Among the MMPs here tested (MMP-2 and MMP-9), only MMP-2 was affected by HT. We cannot explain the lack of effect by HT on MMP-9 expression in our experimental condition. TNF-α caused a strong induction of MMP-9 mRNA levels, and it is possible that different treatment time and/or concentrations of HT are necessary to disclose any effect on MMP-9 expression. MMP-2 (and MMP-9) expression and secretion have been found in human AT [75], and in the AT of obese mice [76]. Moreover, an increase of MMP-2 and a decrease of tissue inhibitor of MMPs type 1 (TIMP-1) during adipocyte differentiation have been demonstrated [77]. Blockage of MMPs, including MMP-2, with pharmacological inhibitors or neutralizing antibodies reduced adipocyte differentiation [75], fat pad weight and adipocyte size [76]. In addition, mice lacking MMP-2 [78], but not MMP9 [79], are also resistant to obesity induced by high-fat diet. Therefore, MMP-2 may play a role in AT expansion, through the degradation of the extracellular matrix and basement membrane components, the activation of latent growth factors, and the stimulation of angiogenesis and adipogenesis.

AT expansion also requires the formation and function of new blood vessels, through the process of angiogenesis, where a critical regulator is VEGF-A [80]. VEGF, inducible by hypoxia and inflammatory signals, is highly expressed in AT and in adipocytes, where its expression increases during adipocyte differentiation [80]. A reciprocal regulation between adipocyte differentiation and angiogenesis has been proved, whereby inhibition of adipocyte differentiation also abrogated fat tissue and angiogenesis, and inhibition of angiogenesis (by blocking VEGF receptor-2 on the vascular endothelium) reduced tissue growth, but also preadipocyte differentiation [81]. AT-specific VEGF repression has recently reported to lead to adipose browning, increased energy consumption, and reduced body weight [82]. Therefore, disruption of AT neovascularization, also targeting adipocytes, might be potentially useful for obesity prevention. Accordingly, curcumin, the major polyphenol in turmeric spice, suppresses adipose accumulation through its effects on angiogenesis and adipogenesis [83]. For the first time, we here found a significant downregulation of stimulated VEGF mRNA and protein levels by HT in human adipocytes. HT ability to regulate VEGF expression has been confirmed in other cell types, including cancer cells [84] and human chondrocytes [85]. We have previously demonstrated anti-angiogenic effects of HT [25] and EVOO extracts [86] in endothelial cells stimulated with VEGF, through the modulation of COX-2, MMP-2 and MMP-9, accompanied by reduced ROS production. Although requiring further confirmation, a similar anti-angiogenic effect might be exerted by HT in AT, possibly mediated by the downregulation of adipocyte production of VEGF and proteolytic enzymes. As the modulation of angiogenesis and proteolytic systems have the potential to impair the development of obesity, the inhibition of VEGF and proteolytic enzymes by HT may contribute to its anti-obesity effects documented in vivo.

HT also reduced TNF-α-stimulated mRNA and protein expression of COX-2 and the related production of PGE_2_. COX-2 is a proinflammatory enzyme playing a key role in chronic inflammatory diseases, and catalyzes the committed step in prostanoid biosynthesis, converting free arachidonic acid into the PG precursors, followed by the formation of prostanoids, including prostacyclin (PGI_2_) and PGE_2_. COX-2 also possesses pro-angiogenic and pro-remodeling properties, being able to induce MMP production [87]. Recently, a role for COX-2 in AT inflammation in obesity has been demonstrated: COX-2 expression is high in the AT, increases in human adipocytes under obesity, and correlates with BMI in humans [88]. Using selective inhibitors in animal models of obesity and in cultured adipocytes, AT COX-2 and its downstream product PGE_2_ have been seen to contribute in mediating inflammation in visceral fat (increased NF-κB activity, MCP-1, TNF-α, leptin and ROS production, macrophage infiltration and decreased adiponectin) accompanied by the development of systemic IR and fatty liver [88,89]. Besides hypertrophy and hypoxia [88], we showed that TNF-α significantly upregulated COX-2 gene expression in adipocytes, as previously reported [90]. COX-2 modulation by HT is consistent with similar results obtained in endothelial cells [25,91], and monocytes/macrophages [27,92] under inflammatory condition, although some studies have used very high concentrations (≥50 µmol/L) [91,92]. The adipocyte COX-2/PGE_2_ axis could be a novel therapeutic target of HT health properties against obesity-associated IR and T2DM.

Collectively, these data corroborate and further expand the human reports of inhibitory effects by polyphenol-rich EVOO consumption, within or out of the frame of the Mediterranean diet, on plasma levels [93,94,95] as well as monocyte [93,96,97,98,99] and endothelial cell [100] gene expression of pro-inflammatory biomarkers, such as cytokines, chemokines, adhesion molecules (e.g., IL-1β, IL-6, MCP-1, sICAM-1, COX-2, MMPs) in healthy subjects and in people at high CV risk including overweight/obese or metabolic syndrome subjects. This suggests a global anti-inflammatory nutrigenomic effect by HT that may be also contributed by the regulation of adipocyte function, as here demonstrated. However, at variance with the above-mentioned human studies it should be acknowledged that the present data are obtained in cultured cells and not in humans, and with a single EVOO component, HT, thus not capturing potential synergistic actions occurring with food or dietary patterns. 

In the present study, we also showed that HT reverted the TNF-α-induced a downregulation of PGC-1α, eNOS, and GLUT4 in SBGS adipocytes. PGC-1α is a key transcriptional regulator of mitochondrial biogenesis, metabolic gene expression (e.g., GLUT-4) fatty acid oxidation, thermogenesis and browning of the AT, and is reduced in the AT of insulin resistant and obese subjects [101]. Targeting its activity has become therefore a prospect for obesity therapies. The PGC-1α regulation by HT, accompanied by improvement of mitochondrial function, energy expenditure and anti-adipogenic effects, had been already reported by Hao et al. [34] in 3T3L-1 adipocytes, and by Stefanon et al. [33] in human omental adipocytes under basal condition, as well as by Shen et al. [49] in the AT of obese mice treated with an olive leaf extract rich in HT. The observed PGC-1α mRNA upregulation by HT in inflammatory condition is accompanied by the recovery of mRNA expression of the insulin-responsive glucose transporter GLUT-4 in the present study, and is concordant with the upregulation of thermogenic UCP-1 and UCP-2, as reported by others [49,102], indicative of browning activity of white AT. It remains to verify whether GLUT-4 upregulation by HT would also be accompanied by improved glucose uptake under inflammatory condition.

HT may interfere with the TNF-α-induced downregulation of PGC-1α acting on different molecular switches upstream of PGC-1α expression and/or activation. One of these is AMP-activated protein kinase (AMPK), a sensor of cell energy status, that can stimulate the expression of PGC-1α, or increase PGC-1α activity directly by inducing its phosphorylation or indirectly by increasing SIRT1, the major NAD^+^-dependent deacetylase controlling PGC-1α activity [103]. HT has been reported to activate AMPK [34], through mechanisms likely including adiponectin [35] and/or SIRT1 expression upregulation [33,49].

Another signaling node of the AMPK/PGC-1α axis is NO, a reactive nitrogen molecule that is formed enzymatically by nitric oxide synthase (NOS), via the conversion of L-arginine to L-citrulline. Beyond the vasodilator and anti-atherogenic properties, eNOS-dependent NO, at physiological concentrations, positively impacts mitochondrial function and is required for mitochondrial biogenesis, brown AT signaling, and energy expenditure, displaying insulin sensitizing and anti-obesity effects [104]. Indeed, a positive feedback loop between NO production and AMPK activation has been demonstrated, that ultimately induces the expression/activity of PGC-1α [105]. Human and animal IR and obesity are associated with reduced NO bioavailability [106]. Overexpression of eNOS prevented the weight gain and the appearance of metabolic syndrome features in a mouse model of diet-induced obesity [107]. Conversely, eNOS deficient mice displayed reduced mitochondrial biogenesis and function, hyperinsulinemia and IR, and increased abdominal fat accumulation and body weight compared with wild-type animals [104]. Previous studies found that EVOO polyphenols enhanced NO by activating eNOS in the endothelium, decreasing plasma asymmetric dimethylarginine (ADMA) (an inhibitor of NO synthesis), in concomitance with improvement in vascular reactivity, reduced markers of oxidative stress and vascular inflammation (oxidized LDL, adhesion molecules, CRP, etc.), that impairs NO synthesis and bioavailability [108,109,110,111]. Although eNOS is primarily expressed in endothelial cells, it has also been found in adipocytes [112], where inflammatory stimuli, including TNF-α, can downregulate its expression and abundance, thus impairing mitochondrial function and energy metabolism. We here provided the first demonstration of an improvement of eNOS in dysfunctional adipocytes. Interestingly, eNOS in AT is also involved in the production of adiponectin [113], which we have previously shown to be improved by HT [35]. Collectively, the HT-induced restoration of eNOS/AMPK/PGC-1α levels under inflammatory condition may contribute to the metabolic benefit of HT.

ROS production increases in the AT during obesity, and is implicated in the development of whole body IR, adipocyte inflammation with adipokine dysregulation, and the recruitment of macrophages to the AT [2,114]. Increased ROS production in accumulated fat is linked to activated NADPH oxidase pathway, mitochondrial dysfunction, uncoupled eNOS (in the absence of sufficient amount of cofactors or substrates), and to impaired antioxidant defense, as indicated by reduced expression and activity of antioxidant enzymes such as SOD and GPX, important for detoxifying superoxide anion and hydrogen peroxide (as well as organic hydroperoxides), respectively [2,114]. Therefore, we examined the effects of HT on ROS levels and the mRNA expression of SOD and GPX in adipocytes stimulated with TNF-α. We found that HT attenuated ROS overproduction induced by TNF-α. Interestingly, in agreement with ROS induction, TNF-α caused a remarkable increase in the mRNA expression of the antioxidant enzymes SOD and GPX compared with unstimulated control, likely attributable to an adaptive defense response to restore the oxidant/antioxidant balance. The decrease in SOD and GPX mRNA levels observed after HT plus TNF-α compared with TNF-α alone is not surprising, as may indicate that oxidative stress can be effectively blunted by HT, and that HT-treated cells might no longer require high levels of antioxidant enzymes. HT and other EVOO polyphenols have been shown to be effective in counteracting tissue and systemic oxidative stress [30,115,116,117], through direct ROS scavenging, improvement of mitochondrial function, reducing endoplasmic reticulum stress, and upregulating antioxidant/detoxifying enzymes, such as SOD, GPX, catalase, heme oxigenase-1 in vitro and in vivo, by stimulating key transcription factors such as nuclear erythroid 2-related factor-2 (Nrf-2) and FOXO3a, in different cell types and tissues [23,102,118,119]. With specific regard to AT, our findings are in line with the reported ability of HT to blunt the H_2_O_2_-induced GSH/GSSG alteration indicative of oxidative stress in adipocytes, and to positively modulate the GSH-driven antioxidant enzymatic machinery, including also SOD and GPX, in AT [23]. On the other hand, an effect of HT on adipocyte NADPH activity and/or expression, a main source of ROS, cannot be ruled out because an EVOO polyphenol extract and EVOO serum metabolites downregulate NADPH activity and/or expression, as demonstrated in vascular tissue [86,116].

Many adipokines (cytokines, chemokines), adhesion molecules and other adipocyte dysfunction-related markers (COX-2, MMPs, VEGF) here assessed are regulated by NF-κB, a master transcriptional factor of inflammatory responses [6,120]. A number of signals, including those associated with obesity, such as cytokines like TNF-α and Toll-like receptor (TLR) agonists of pathogenic or dietary origin (e.g., in the presence of excess free fatty acids), can boost NF-κB pathways. Furthermore, NF-κB activation is regulated by ROS-sensitive pathways, being hence targetable by antioxidants. NF-κB has been increasingly implicated in the initiation and progression of metabolic diseases, connecting inflammation and dysmetabolism in the AT [40]. We therefore determined whether HT can blunt the transcriptional pro-inflammatory response in adipocytes by attenuating the activation of NF-κB. In accordance with HT ability to inhibit NF-κB in other cell types, including endothelial cells [74] and monocytes/macrophages [24,121], HT decreases the DNA binding activity of the NF-κB p65 subunit, an indicator of NF-κB activation, in nuclear extracts from human adipocytes. Further studies are needed to determine the mechanisms underlying HT-mediated attenuation of NF-κB in adipocytes, but some modes of action by HT may be inferred, some of which presented here, including the inhibition of oxidative stress, as well as the upregulation of PGC-1α, that has been found to repress pro-inflammatory responses by inhibiting NF-κB signaling and to be suppressed by NF-κB itself [122], providing regulatory interlinks between energy homeostasis and inflammation. Notably, PGC-1α is a coactivator of PPARγ, which negatively affects NF-κB signaling [123] and is upregulated by HT in TNF-α-stimulated SGBS adipocytes [35]. The induction of SIRT-1 by HT [33] may also lead to NF-κB inhibition by deacetylating the p65 subunit [124].

Another relevant observation of the present study is the ability of HT to limit miRNA expression changes linked to inflammation driven by TNF-α, in both adipocytes and exosomes derived from adipocyte supernatants. In particular, we documented, for the first time, that part of the anti-inflammatory role of HT may be attributable to its ability to restore the expression of inflammatory miRNAs in TNF-α-treated adipocytes, i.e. downregulating miR-155 and miR-34a and upregulating let-7c. miR-155 is one of the most dynamically regulated and multifunctional microRNAs, which has been associated with the regulation of immune-related processes, including innate immunity, macrophages inflammatory activation, B-cell and T-cell differentiation, with an impact on cancer [125] and atherosclerosis [126]. miR-155 had been reported to be increased by TNF-α-stimulated adipocytes via NF-κB activation [16], and in subcutaneous AT biopsies of obese subjects, where it mediates the induction of an inflammatory state by regulating genes related to NF-κB activation, toll-like receptor signaling, and chemokines [16]. Concordantly, the deletion of miR-155 in mice prevented diet-induced obesity, improved insulin sensitivity, and abrogated adipocyte hypertrophy and AT inflammation [127]. miR-155 has also been seen to promote inflammatory activation of macrophages by repressing B-cell leukemia/lymphoma (BCL) 6, a negative regulator of NF-κB signaling, thus promoting atherosclerosis [126]. In line with our data, preincubation with another natural compound, 1,25(OH)2 vitamin D, limited miR-155 levels in 3T3-L1 murine adipocytes incubated with TNF-α [128]. Furthermore, the decreased levels of miR-34a induced by HT are in line with the pro-inflammatory role of this miRNA. miR-34a expression in visceral fat increased with the development of obesity and is closely associated with IR and inflammation [14]. Mechanistically, miR-34a is able to increase NF-κB signaling by directly targeting SIRT-1 [129]. Moreover, adipocyte-secreted exosomal miR-34a drives macrophage M1 polarization by targeting Kruppel-like factor (KLF) 4 [14]. Finally, recent studies have shown the anti-inflammatory role of let-7c, which has been reported to promote the skewing of macrophages toward the M2 phenotype, and to suppress NF-κB as well as STAT3 signaling, thus attenuating the expression of downstream pro-inflammatory cytokines [18,130]. Additionally, according to miRTarBase database of experimentally validated microRNA-target interactions [131], COX-2 and IL-6 are validated targets of let-7c. Thus, it appears that HT may directly reduce the TNF-α-stimulated IL-6 and COX-2 gene expression by increasing let-7c. miRWalk database [132] confirms IL-6 as a validated target and COX-2 as a predicted target of let-7c. Interestingly, another member of the TNF-α protein family, the TNF-related apoptosis-inducing ligand (TRAIL-R2), which has been recently involved in obesity and AT inflammation [133], is targeted by let-7c, thus the let-7c-mediated anti-inflammatory action of HT might be even wider and deserves further investigation.

The involvement of NF-κB activity, inhibited by HT, in the induction of miR-155 [16], miR-34a [134], and the suppression of let-7c [135] may mediate the HT modulatory effect on these miRNAs. As for other polyphenols [136], previous studies have reported miRNA modulation by HT both in vitro with implications in oxidative stress (miR-9 targeting SIRT-1) [137] and inflammation (miR-146 linked to Nrf-2 pathway) [138], and in vivo, where miRNAs involved in metabolic, redox and transport processes were influenced by HT in the mice intestine and other tissues [139]. In agreement with our results, a HT sulfate metabolite recovered the IL-1β-induced downregulation of let-7 miRNA in preventing endothelial dysfunction [140]. Accordingly, acute intake of high polyphenol-EVOO by healthy subjects modulated the expression of miRNAs involved in metabolic, inflammatory and redox pathways [141].

**Figure 10:**
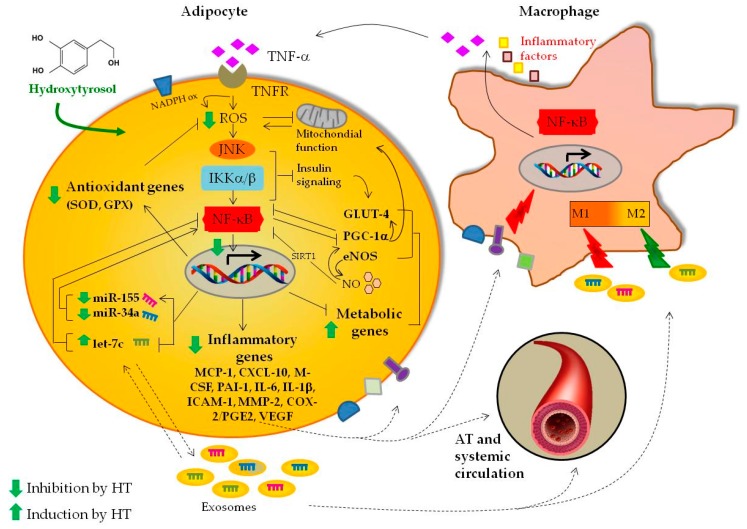
Schematic summary of the modulation by HT of TNF-α-induced gene and miRNA expression in adipocytes. Upon binding to the cognate receptor, TNF-α, which is mostly produced by the adipose tissue (AT) inflammatory macrophages (M1 polarized), triggers a cascade of signaling events involving the overproduction of ROS (by dysfunctional mitochondria and other sources such as NADPH oxidase), as well as the activation of MAPK and IKK/NF-κB pathways, culminating in the upregulation of pro-inflammatory genes and miRNAs (miRs) including miR-155 and miR-34a, and the downregulation of anti-adipogenic metabolic genes (eNOS, PGC-1α, GLUT-4), and anti-inflammatory miRs (let-7c). These deleterious gene expression changes may act in a paracrine and endocrine manner inducing adipocyte dysfunction as well as macrophage recruitment/inflammatory polarization (M1 phenotype). HT inhibits TNF-α-induced oxidative stress and NF-κB activation, and ameliorates the pattern of gene and miRNA expression toward a potential anti-inflammatory, insulin-sensitizing and anti-adipogenic profile. See text for further details. The arrow indicates stimulation. The line indicates inhibition. JNK: c-Jun N-terminal kinase; MAPK: mitogen-activated protein kinase; NADPH ox: NADPH oxidase; TNFR: TNF-α receptor; IKK: IκB kinase.

Considering the regulatory roles exerted by miR-155, miR-34a, and let-7c in both adipocytes and inflammatory cells, the concordant modulation of these inflammation-related miRNAs by HT in both adipocytes and their exosomes suggests that HT may beneficially impact not only directly on adipocyte dysfunction, but also indirectly on pro-inflammatory miRNA-mediated paracrine and endocrine cross-talk between adipocyte and macrophages, other cells, and distant organs. An integrative diagram of the proposed effect of HT on gene and miRNA expression under adipocyte dysfunction and inflammation induced by TNF-α is depicted in Figure 10.

The modulation of inflammatory genes and miRNAs expression of adipocytes reported in this study was evident at HT concentrations (1 and 10 μmol/L) comparable to plasma levels attainable after EVOO consumption. Indeed, acute intake of EVOO, at doses (25–40 mL) corresponding to real-life daily OO consumption within traditional Mediterranean diets, resulted in a plasma concentration range of around 50 nmol/L [142] to 20 μmol/L [68,143] of HT (and/or its metabolites), depending on the OO polyphenol content. Although ingested HT undergoes extensive hepatic/intestinal and microbial biotransformation, the question whether free HT per se or also its metabolites are active has not been resolved yet. Emerging studies suggest that the health benefits of VOO polyphenols may be attributed either to the phase II metabolites or to free HT generated at the cellular level by enzymatic deconjugation [43]. Therefore, the effect of HT metabolites on adipocytes and AT biology deserves further verification. 

The present study has several limitations. First, we tested a single polyphenol as commercially available and not isolated from EVOO. Second, we used a simplified cell model resembling adipocyte dysfunction under obesity. Therefore, the potential synergistic effects occurring in the food matrix among different polyphenols as well as the bioactivity of the major in vivo bioavailable forms could not be evaluated. For these reasons, results should be interpreted with cautions and the transferability to humans requires further studies in human subjects in real life condition. 

## 5. Conclusions

The present study is the first demonstration of a beneficial integrated modulation by HT of the altered pattern of gene expression (transcripts and miRNAs) related to inflammation and metabolism in inflamed human adipocytes. The precise mode of action of HT and the functional implications remain to be determined, but our in vitro results provide novel basic knowledge about the cardiometabolic benefit ascribed to EVOO and the Mediterranean diet, as well as to HT as a basic component of nutraceutical formulations. Further studies are warranted to recapitulate these findings in animal models and clinical settings.
